# Fewest switches surface hopping with Baeck-An couplings

**DOI:** 10.12688/openreseurope.13624.1

**Published:** 2021-05-17

**Authors:** Mariana T. do Casal, Josene M. Toldo, Max Pinheiro Jr, Mario Barbatti

**Affiliations:** 1 Aix Marseille University, CNRS, ICR, Marseille, France

**Keywords:** Computational chemistry, Ultrafast dynamics, Nonadiabatic dynamics, Surface hopping, Fewest Switches, Ethylene, Fulvene

## Abstract

In the Baeck-An (BA) approximation, first-order nonadiabatic coupling vectors are given in terms of adiabatic energy gaps and the second derivative of the gaps with respect to the coupling coordinate. In this paper, a time-dependent (TD) BA approximation is derived, where the couplings are computed from the energy gaps and their second time-derivatives. TD-BA couplings can be directly used in fewest switches surface hopping, enabling nonadiabatic dynamics with any electronic structure methods able to provide excitation energies and energy gradients. Test results of surface hopping with TD-BA couplings for ethylene and fulvene show that the TD-BA approximation delivers a qualitatively correct picture of the dynamics and a semiquantitative agreement with reference data computed with exact couplings. Nevertheless, TD-BA does not perform well in situations conjugating strong couplings and small velocities. Considered the uncertainties in the method, TD-BA couplings could be a competitive approach for inexpensive, exploratory dynamics with a small trajectories ensemble. We also assessed the potential use of TD-BA couplings for surface hopping dynamics with time-dependent density functional theory (TDDFT), but the results are not encouraging due to singlet instabilities near the crossing seam with the ground state.

## Plain language summary

When a molecule absorbs light, its electrons are excited. The molecular geometry distorts until the excess of energy dissipates as heat or is reemitted as light. It is possible to simulate using a computer how a molecule reacts to light excitation. Still, such dynamics simulations involve complex and slow quantum-mechanical methods. In this work, we present a new approach to compute one of the quantities needed for the simulations, the nonadiabatic couplings. Based on a new theory proposed by Baeck and An, our method (we call it TD-BA) gets the couplings from electronic energies and their variations with time. Because these quantities are always available during dynamics simulations, TD-BA couplings are much simpler and faster to compute than usual couplings. We tested our TD-BA method for the simulation of two molecules, ethylene and fulvene. The results show that TD-BA does a good job predicting the dynamics of these molecules. 

## I. Introduction

Surface hopping has become one of the main tools for nonadiabatic dynamics simulations of photoexcited molecules ^1, 2^. Its popularity stems from its underlying independent trajectory and local approximations, requiring electronic quantities computed only at the nuclear geometry of classical trajectories. Moreover, surface hopping is usually done in adiabatic representation, avoiding cumbersome diabatization procedures usually required for wavepacket propagation. Thus, surface hopping is commonly implemented to work with on-the-fly calculations of electronic properties (energies, energy gradients, and couplings) obtained with many different quantum chemical methods ^3– 9^, and it requires neither pre-calculated nor modeled global potential energy surfaces.

The primary constraint for interfacing surface hopping to a new quantum chemical method or program is to get the first-order nonadiabatic coupling vectors $h_{JL}=\langle J|\frac{\partial }{\partial R}L\rangle$ between adiabatic electronic states *J* and *L*. These quantities are not as widespread in quantum-chemical packages as excited-state energy gradients for instance, though the situation has improved in the last two decades ^10– 12^. Alternatively, the coupling vectors can also be obtained from an approximation involving potential energy Hessian matrices ^13, 14^. Moreover, there are surface hopping variants that do not even require explicit nonadiabatic coupling calculations, as is the case of the Zhu-Nakamura ^15, 16^ and Belyaev-Lebedev ^17– 19^ surface hopping approaches. The former estimates nonadiabatic events from minimum energy gaps and energy gradients around this minimum, while the latter does from minimum energy gaps and second time-derivative of the gap at the minimum gap. The popular fewest switches surface hopping (FSSH) ^20^ requires nonadiabatic coupling vectors. Nonetheless, because these vectors are always projected on the velocities within the formalism, they can be replaced by time-derivative couplings $\sigma_{JL}\equiv \langle J|\frac{\partial }{\partial t}L\rangle =v\cdot h_{JL}$ , which are much simpler to implement than nonadiabatic coupling vectors. In general, the calculation of time-derivative couplings follows the Hammes-Schiffer-Tully prescription ^21^, and they are obtained from wavefunction overlaps ^22– 24^. Alternatively, the coupling vector calculation can be avoided entirely in FSSH, by employing the local diabatization formalism ^25^, which is also based on wavefunction overlaps.

All these models possess algorithmic limitations for their universal adoption. The Hessian-based nonadiabatic coupling approximation is too costly for on-the-fly dynamics, although it has been used with machine-learning (ML) nonadiabatic dynamics ^26^. Zhu-Nakamura and Belyaev-Lebedev approaches require determination of the diabatic crossing point, which, translated into adiabatic representation, means that the hopping at time *t* depends not only on the previous timesteps but also on the future of the trajectory. (These methods are usually implemented in terms of a three-point approximation ^15, 17– 19^, evaluating the hopping at *t* from information computed at *t* – Δ *t*, *t*, and *t* + Δ *t*.) On the other hand, the wavefunction overlaps required by time-derivative coupling and local diabatization approaches boil down to nonorthogonal atomic-orbital overlap matrices, demanding lengthy algorithmic intervention whenever a new method or program needs to be interfaced to surface hopping.

Recently, Baeck and An ^27^ conjectured that nonadiabatic couplings near an energy crossing point could be estimated from the simple 1D model

$$h_{JL}\left(Q\right)\approx \frac{\text{sgn}\left(\text{Δ}E_{JL}\left(Q\right)\right)}{2}\sqrt{\frac{1}{\text{Δ}E_{JL}\left(Q\right)}\frac{\partial^{2}\text{Δ}E_{JL}\left(Q\right)}{\partial Q^{2}}}\;Q\approx Q_{c}\;(1)$$

involving only the energy gap Δ *E _JL_* = *E _J_* – *E _L_* and the second derivative of this gap with respect to the coupling direction *Q*. Details of their model are surveyed in Section II. Baeck and An’s numerical tests for different molecules have indeed shown an outstanding agreement between this simple approximation and the exact nonadiabatic coupling ^27, 28^. Our goal in this paper is to expand and explore the Baeck-An (BA) model to be used in surface hopping dynamics.

Baeck and An pointed out two potential limitations of their model ^27^. First, it is strictly valid for molecular configurations coupling only two states. Second, the model is restricted to a single degree of freedom. When using their model for dynamics, there is nothing that we can do about the first limitation but to hope that two-state coupling dominates the nonadiabatic interactions. However, concerning the second limitation, it is fortunate that FSSH does not require the full nonadiabatic coupling vector but only its projection on the velocity direction. As we shall discuss, we can explore this feature to expand the 1D BA model to multidimensional dynamics, using a time-dependent (TD) BA model.

The TD-BA model is not supposed to replace the exact calculation of nonadiabatic couplings in surface hopping. It can be, however, an alternative approach to be used with electronic structure methods for which nonadiabatic couplings are still unavailable or are too computationally expensive to be computed on-the-fly. The TD-BA model can also be used as an inexpensive diagnostic of nonadiabatic interactions during exploratory dynamics, triggering higher-level calculations. Another potential use of the TD-BA model is in ML. Several authors have reported difficulties in the ML-prediction of nonadiabatic couplings ^29– 31^. Their narrow functional shapes require too large training sets and active learning, being the main challenge for developing ML nonadiabatic dynamics. The TD-BA model, requiring only predicting energy gaps, may open a new avenue to overcome these problems.

**Figure 1.**
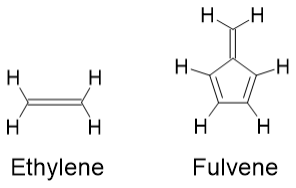
Molecular structure of ethylene and fulvene.

In this work we show how TD-BA couplings can be seamlessly integrated into FSSH. We tested it by performing decoherence-corrected (DC) FSSH dynamics for ethylene and fulvene ( Figure 1) using TD-BA couplings and comparing the results to DC-FSSH dynamics of these molecules using full nonadiabatic coupling vectors. Dynamics of both systems have been extensively studied before, and, recently, Ibele and Curchod ^32^ proposed that they may be considered molecular examples for two of the three basic 1D Tully models ^20^. Ethylene is a crucial prototype ^32^ for internal conversion in various systems as diverse as protonated Schiff bases ^33^, substituted polyenes ^34^, and purine bases ^35^. In turn, fulvene poses the challenge of S _1_↔S _0_ recurrences at an extended crossing seam with both peaked and sloped conical intersections ^36^. The results for both molecules are encouraging, showing that TD-BA-based dynamics delivers a semiquantitative agreement with reference datasets computed with exact couplings.

## II. The BA model for nonadiabatic couplings

Baeck and An have shown that the nonadiabatic coupling maximum for a two-states system can be approximated as ^27^

$$h_{JL}\left(Q_{c}\right)\equiv {\langle J|\frac{\partial L}{\partial Q}\rangle |}_{Q_{c}}\approx \frac{\text{sgn}\left(\text{Δ}E_{JL}\left(Q_{c}\right)\right)}{2}\sqrt{\frac{1}{\text{Δ}E_{JL}\left(Q_{c}\right)}{\frac{\partial^{2}\text{Δ}E_{JL}}{\partial Q^{2}}|}_{Q_{c}}}\;(2)$$

where *Q* is a one-dimensional coordinate coupling the adiabatic states *J* and *L*. *Q _c_* is the geometry where the magnitude of the coupling is the largest. This expression arises from assuming that the nonadiabatic coupling can be represented as a Lorenz function of *Q*,

$$\;h_{JL}\left(Q\right)=\frac{1}{2}\frac{\kappa \left(\frac{{\text{Δ}}_{c}}{2}\right)}{\left({\left(\frac{{\text{Δ}}_{c}}{2}\right)}^{2}+\kappa^{2}{\left(Q-Q_{c}\right)}^{2}\right)}\;(3)$$

where *κ*, the coupling constant, and Δ _*c*_, the adiabatic energy gap at *Q _c_*, are constants. At *Q _c_*, the maximum nonadiabatic coupling is simply

$$\;h_{JL}\left(Q_{c}\right)=\frac{\kappa }{{\text{Δ}}_{c}}\;(4)$$

Δ _*c*_, which by construction corresponds to twice the diabatic coupling, is given as the adiabatic energy gap between *J* and *L* at *Q _c_*

$$\;{\text{Δ}}_{c}\equiv \text{Δ}E_{JL}\left(Q_{c}\right)\;(5)$$

In turn, to get the coupling constant *κ*, Baeck and An explore the similarities between their model and the linear vibronic coupling (LVC) model ^37^. While in LVC, Köppel, Gronki, and Mahapatra propose to compute *κ* from

$$\;\kappa ={\left[\frac{1}{8}\frac{\partial^{2}\text{Δ}E_{JL}^{2}}{\partial Q^{2}}\right]}_{Q_{c}}^{1/2}\;(6)$$

Baeck and An propose the expression

$$\;\begin{array}{l} \kappa ={\left[\frac{1}{8}\frac{\partial^{2}\text{Δ}E_{JL}^{2}}{\partial Q^{2}}-\frac{1}{4}{\left(\frac{\partial \text{Δ}E_{JL}}{\partial Q}\right)}^{2}\right]}_{Q_{c}}^{1/2}\\ \;=\frac{1}{2}{\left[\text{Δ}E_{JL}\frac{\partial^{2}\text{Δ}E_{JL}}{\partial Q^{2}}\right]}_{Q_{c}}^{1/2} \end{array}\;(7)$$

which, compared to LVC, equivales to introduce an asymmetric linear term in *Q* in the expansion of Δ *E _JL_*. This term aims to make the model more flexible so that the minimum of Δ *E _JL_* does not necessarily coincide with *Q _c_*.

Finally, the nonadiabatic coupling maximum in Equation (2) is obtained by replacing *κ* given in Equation (7) into Equation (4).

Baeck and An also conjectured that the functional form of Equation (2) extended to an arbitrary point *Q* should describe the nonadiabatic coupling also in the region near *Q _c_*. This is the source of Equation (1).

## III. TD-BA couplings

To employ the BA model in surface hopping, we start by writing their expression as a vector in the unitary direction of **Q**

$$\;h_{JL}\left(R\right)\approx \frac{\text{sgn}\left(\text{Δ}E_{JL}\left(R\right)\right)}{2}\sqrt{\frac{1}{\text{Δ}E_{JL}\left(R\right)}\frac{\partial^{2}\text{Δ}E_{JL}\left(R\right)}{\partial Q^{2}}}\hat{Q}\;R\approx R_{c}\;(8)$$

where **R** is the full-dimensional molecular geometry, which has a crossing at **R**_*c*_. In the spirit of the Baeck-An 1D-approximation, Equation (8) assumes that the only non-null energy Hessian component is that one in the *Q* direction.

Using the chain rule, we can rewrite the Hessian as a time derivative

$$\;\frac{\partial^{2}\text{Δ}E_{JL}}{\partial Q^{2}}=\frac{1}{v_{Q}^{2}}\frac{d^{2}\text{Δ}E_{JL}}{dt^{2}}\;(9)$$

where *v _Q_* is the projection of the nuclear velocity on $\hat{Q}$ . Replacing Equation (9) in Equation (8) yields the time-dependent version of the Baeck-An nonadiabatic coupling

$$h_{JL}\left(t\right)\approx \frac{\text{sgn}\left(\text{Δ}E_{JL}\left(t\right)\right)}{2v_{Q}}\sqrt{\frac{1}{\text{Δ}E_{JL}\left(t\right)}\frac{d^{2}\text{Δ}E_{JL}\left(t\right)}{dt^{2}}}\hat{Q}\;t\approx t_{c}\;(10)$$

which implicitly assumes that $\hat{Q}$ direction is constant. In the previous expression, *t _c_* is the time at which the molecule reaches **R**_*c*_.

In FSSH ^20^, the quantity of interest is the time-derivative coupling $\sigma_{JL}\equiv \langle J|\frac{d}{dt}L\rangle =v\cdot h_{JL}$ (see Section IV), which using Equation (10) simplifies to

$$\;\sigma_{JL}\approx v_{Q}h_{JL}=\frac{\text{sgn}\left(\text{Δ}E_{JL}\right)}{2}\sqrt{\frac{1}{\text{Δ}E_{JL}}\frac{d^{2}\text{Δ}E_{JL}}{dt^{2}}}\;(11)$$

Note that all references to *Q* cancel out in Equation (11).

To enforce the condition *t* ≈ *t _c_*, we notice that whenever the molecule moves near a minimum of Δ *E _JL_*, the square-root argument in Equation (11) must be positive. Thus, we assume that the coupling is null for negative arguments, and the TD-BA nonadiabatic coupling becomes

$$\sigma_{JL}\equiv v\cdot h_{JL}\approx \{\begin{array}{cc} \frac{\text{sgn}\left(\text{Δ}E_{JL}\right)}{2}\sqrt{\frac{1}{\text{Δ}E_{JL}}\frac{d^{2}\text{Δ}E_{JL}}{dt^{2}}} & \text{if}\;\frac{1}{\text{Δ}E_{JL}}\frac{d^{2}\text{Δ}E_{JL}}{dt^{2}}>0\\ 0 & \text{if}\;\frac{1}{\text{Δ}E_{JL}}\frac{d^{2}\text{Δ}E_{JL}}{dt^{2}}\leq 0 \end{array}\;(12)$$

Although the conditions in Equation (12) are not sufficient to detect *t* ≈ *t _c_* (the square root argument may still be positive away from the minimum gap), they make a good job screening the relevant crossing regions during dynamics, as our tests show.

## IV. Using the TD-BA model in surface hopping

The FSSH hopping probability from *L* to *J* is computed as ^20^

$$\;P_{L\to J}\left(t\right)=\text{max}\left[0,\frac{-2\text{Δ}\tau }{{\left|c_{L}\left(t\right)\right|}^{2}}\sigma_{LJ}\left(t\right)\text{Re}\left(c_{J}\left(t\right)c_{L}^{*}\left(t\right)\right)\right]\;(13)$$

where *σ _LJ_* is the time derivative coupling given by the TD-BA approximation in Equation (12), and the complex coefficients **c** are the solution of the locally-approximated time-dependent electronic Schrödinger equation

$$\;\frac{dc_{J}}{dt}=-\sum_{K}\left(\frac{i}{\hbar }E_{J}+\sigma_{JK}\right)c_{K}\;(14)$$

Δ *τ* in Equation (13) is the timestep for integrating Equation (14). It is, in general, much smaller than Δ *t*, the timestep to integrate the classical equations of motion.

As usual, the FSSH over-coherence should be corrected and, here, we adopted the simplified decay of mixing ^38^.

Because the **h** direction is unknown, the velocity adjustment after hopping should be made in the direction of the linear momentum **p** ^39^.

## V. Computational details and datasets

We have used ethylene and fulvene ( Figure 1) dynamics to test the method. All calculations were done with the multiconfigurational self-consistent field (MCSCF) method state-averaged over three states for ethylene and two states for fulvene. Details on the computation level of ethylene are discussed in Ref. 39 In brief, the active space was composed of five separate complete active subspaces: the first subspace contained four electrons and four orbitals (π, σ, π*, σ*), and each of the other four subspaces contained two electrons in two orbitals (σ, σ*). Thus, the active space can be denoted as [CAS(4,4) ⊕ 4×CAS(2,2)]. For fulvene, we adopted a standard CAS(6,6) complete active space. The 6-31G(d) basis set ^40^ was used in both cases.

Dynamics was simulated with decoherence-corrected ^38^ fewest switches surface hopping ^20^ for a maximum of 200 fs for ethylene and 60 fs for fulvene. The classical equation were integrated with Δ *t* = 0.1 fs steps. The quantum equations were integrated with Δ *τ* = 0.005 fs steps, using interpolated electronic quantities between classical steps. Decoherence corrections were applied with the standard 0.1 au parameter ^38^.

For ethylene, one set of 500 trajectories was run with the TD-BA model. For the analysis, the TD-BA dataset was compared to surface hopping based on exact nonadiabatic coupling vectors with **h**-adjusted and **p**-adjusted velocities. These two datasets, also composed of 500 trajectories each, are the same ones discussed in Ref. 39. For fulvene, we run three datasets of 200 trajectories each, one with the TD-BA model, and other two with exact nonadiabatic coupling vectors using **h**-adjusted and **p**-adjusted velocities.

For both molecules, the TD-BA model was applied with analytical second time derivatives from quadratic regression (Δ *T* = 0.4 fs) and *δη* = 0.1 au. The conditions associated to *δε* and *δϖ* parameters were not applied. See Subsection VI.A for the definition and discussion of these four parameters.

The initial conditions were sampled from a harmonic oscillator Wigner distribution of the nuclei. For ethylene, they were restricted to the 9.30 ± 0.25 eV excitation window ^39^. For fulvene, the initial conditions were restricted to the 4.00 ± 0.34 eV window.

The MCSCF calculations were done with Columbus (version 7, 09-Oct-2020) ^41, 42^. Dynamics was done with Newton-X (version 2.2 build 15) ^7, 43^ interfaced to Columbus. DC-FSSH based on TD-BA couplings with all options discussed in this paper is available in Newton-X version 2.2 build 15 or higher, to be used with any of the electronic structure methods and programs interfaced to Newton-X.

All datasets for ethylene and fulvene, with all Newton-X input and output files, are freely available (see Data availability) ^44– 46^.

Additional TD-BA DC-FSSH calculations were also done with linear-response time-dependent density functional theory (TDDFT) for fulvene and thiophene. Both sets of simulations were performed with the 6-31G(d,p) basis set using the CAM-B3LYP functional ^47^ for fulvene and ωB97XD functional ^48^ for thiophene. The classical equations of motion were integrated with Δ *t* = 0.1 fs timestep for fulvene (maximum of 60 fs) and Δ *t* = 0.5 fs for thiophene (maximum of 300 fs). The quantum equations were integrated with Δ *τ* = Δ *t*/20, using interpolated electronic quantities between classical steps. The initial conditions were sampled from a harmonic oscillator Wigner distribution of the nuclei. For fulvene, they are the same used for complete active space self-consistent field (CASSCF). For thiophene, the initial conditions were restricted to the 6.2 ± 0.5 eV excitation window. The TD-BA model was applied with analytical second time derivatives from quadratic regression (Δ *T* = 0.4 fs) and *δη* = 0.1 au. The conditions associated to *δε* and *εϖ* parameters were not applied. Dynamics were done with Newton-X (version 2.2 build 15) interfaced to Turbomole 7.5 ^49, 50^. GAMESS is a free and open source program that could be used in place of Turbomole to do the TDDFT calculations ^51^.

The statistical analysis followed the protocol proposed in Ref. 39 to compare datasets. For ethylene, mean values and error bars (95% confidence interval) for seven observables (adiabatic-population and oscillator strength time constants and five structural channel yields) were computed from the trajectories. For fulvene, mean values and error bars were computed for six observables (first decay time constant, S _1_ population at 20 fs, S _1_ population at 60 fs, and three structural yields). These quantities were used to calculate the overlap score *λ*_x_ for each observable and the mean overlap score Λ ^(1,2)^ for the average over all *λ*_x_^39^. *λ*_x_ measures the probability that the observable predictions in two datasets overlap (for some confidence interval). In turn, Λ ^(1,2)^ is near one if the predictions of both datasets significantly overlap and zero if they do not.

## VI. Implementation and tests

### A. General aspects

The second time derivative in Equation (12) can be computed numerically. In our implementation, in the first two classical timesteps of the trajectory and after any hopping, the nonadiabatic coupling is assumed to be null. At the third step, the second time derivative is computed by finite differences with the backward *O*(Δ *t*) approximation

$$\frac{d^{2}\text{Δ}E_{JL}\left(t\right)}{dt^{2}}\approx \frac{1}{\text{Δ}t^{2}}\left[\text{Δ}E_{JL}\left(t\right)-2\text{Δ}E_{JL}\left(t-\text{Δ}t\right)\;+\;\text{Δ}E_{JL}\left(t-2\text{Δ}t\right)]\right]\;(15)$$

From the fourth timestep on, we use the backward *O*(Δ *t* ^2^) approximation

$$\;\begin{array}{l} \frac{d^{2}\text{Δ}E_{JL}\left(t\right)}{dt^{2}}\approx \frac{1}{\text{Δ}t^{2}}[2\text{Δ}E_{JL}\left(t\right)-5\text{Δ}E_{JL}\left(t-\text{Δ}t\right)\\ \;+\;4\text{Δ}E_{JL}\left(t-2\Delta t\right)-\text{Δ}E_{JL}\left(t-3\text{Δ}t\right)] \end{array}\;(16)$$

Alternatively, the second time derivative can be evaluated analytically after carrying out a quadratic regression over a sequence of *N _p_* = Δ *T*/Δ *t* points accumulated over a period Δ *T* of a trajectory integrated with timestep Δ *t*. After the regression, the predicted energy gap as a function of time is Δ *E _JL_* ≈ *at ^2^* + *bt* + *c* and the second time derivative is

$$\;\frac{d^{2}\text{Δ}E_{JL}}{dt^{2}}\approx 2a\;(17)$$

Again, the nonadiabatic coupling is assumed to be null in the first two timesteps of the trajectory and after a hopping, while data are accumulated to do the regression.

To reduce the instabilities in the derivatives caused by small energy discontinuities during dynamics (they are widespread in MCSCF propagation ^52^), we adopted three "cleaning" conditions. Thus, in addition to the choice of second time derivative method (and the associated Δ *T* value in the case of using quadratic regression), these conditions introduce three parameters — *δη*, *δε*, and *δϖ* — whose values are discussed later in this section.

The first of such conditions checks the magnitude of the coupling variation in one timestep Δ *t*. If the rate of this variation is larger than a parameter *δη*, the current coupling is discarded in favor of the coupling in the previous timestep:

-
$$\text{if}\;\frac{\left|\sigma_{JL}\left(t\right)\right|-\left|\sigma_{JL}\left(t-\text{Δ}t\right)\right|}{\text{Δ}t}>\delta \eta \;,\;\text{then}\;\sigma_{JL}\left(t\right)=\sigma_{JL}\left(t-\text{Δ}t\right)$$

The second condition eliminates large couplings at energy gaps that are too large. TD-BA couplings are computed only for energy gaps smaller than a threshold *δε* and assumed to be zero otherwise:

-
$$\text{if}\;\left|\text{Δ}E_{JL}\left(t\right)\right|>\text{Δ}\varepsilon \text{,}\;\text{then}\;\sigma_{JL}\left(t\right)=0$$

The third condition avoids large couplings arising from points with large energy gaps but an anomalously large second time-derivative. The coupling is assumed to be null if the product $\left|\text{Δ}E_{JL}\;\text{Δ}E_{JL}^{\prime \prime }\right|$ is larger than an *δϖ* parameter:

-
$$\text{if}\;\left|\text{Δ}E_{JL}\left(t\right)\text{Δ}E_{JL}^{\prime \prime }\left(t\right)\right|>\delta \varpi \;,\;\text{then}\;\sigma_{JL}\left(t\right)=0$$

**Figure 2.**
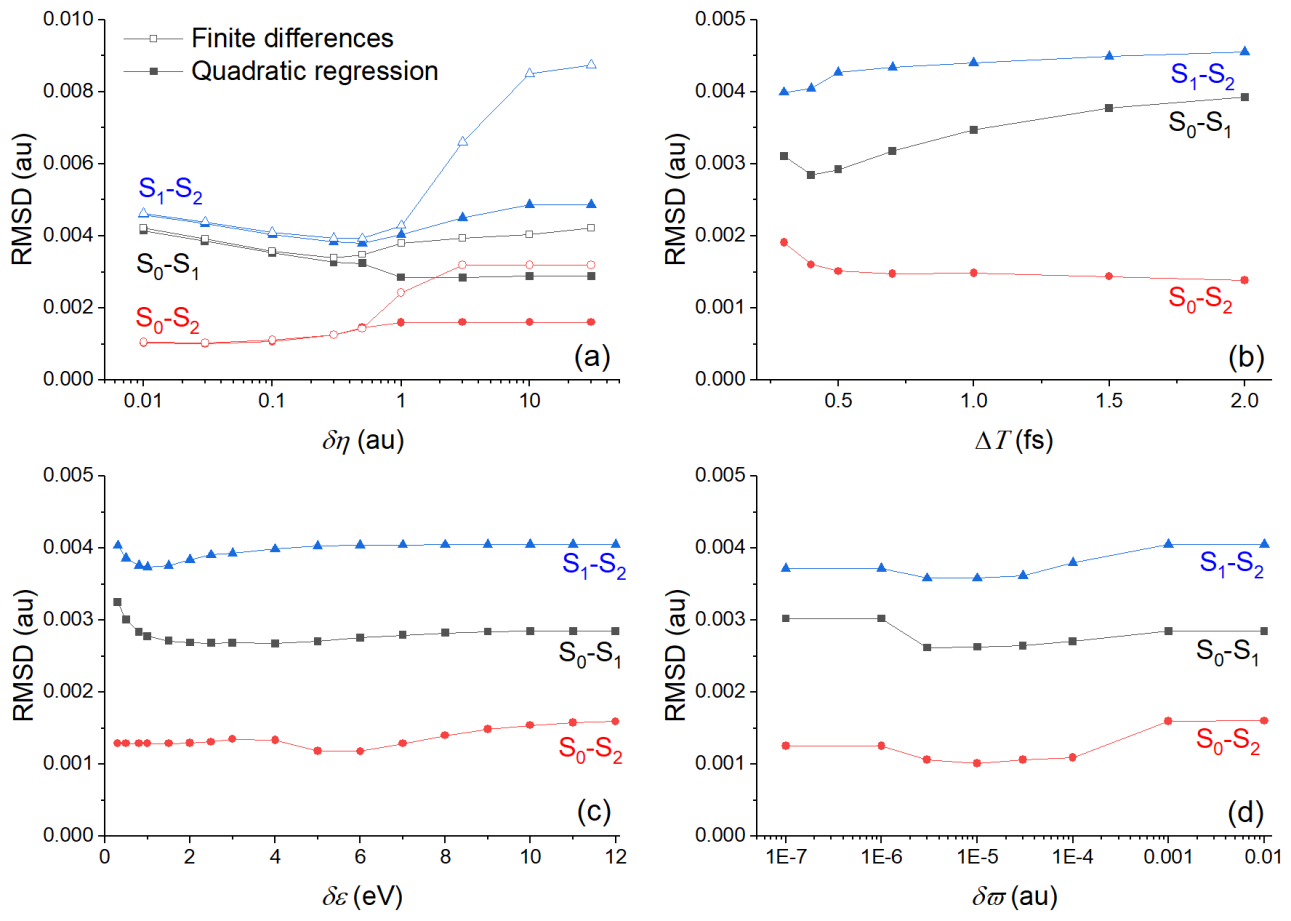
Root mean square deviation (RMSD) between time-dependent Baeck-An (TD-BA) and exact nonadiabatic couplings computed for the ethylene’s **h**-adjusted dataset. ( **a**) RMSD is given for different values of *δη* with analytical (from quadratic regression over Δ *T* = 0.4 fs) and numerical (via finite differences) second time derivatives. The energy gap threshold ( *δε*) is 12 eV and *δϖ* = 0.01 au. ( **b**) RMSD is given for different quadratic regression periods (Δ *T*) for *δη* = 0.1 au, *δε* = 12 eV, and *δϖ* = 0.01 au. ( **c**) RMSD is given for different energy gap threshold ( *δε*) for *δη* = 0.1 au, Δ *T* = 0.4 fs, and *δϖ* = 0.01 au. ( **d**) RMSD is given for different *δϖ* values for *δη* = 0.1 au, Δ *T* = 0.4 fs, and *δε* = 12 eV. Note the log scales in ( **a**) and ( **d**).

Before doing any dynamics with the TD-BA couplings, we investigated the effect of using either method for evaluating second time derivatives and adopting the three cleaning conditions. To do so, we computed TD-BA couplings with different parameters setups for all points of the ethylene’s **h**-adjusted dataset ^39^. The results are summarized in Figure 2. This figure reports the root mean square deviation (RMSD) between the TD-BA coupling *σ _JL_* and the exact ${\hat{\sigma }}_{JL}$ computed for the **h**-adjusted dataset

$$RMSD=\sqrt{\frac{1}{N}\sum_{k=1}^{N_{Traj}}\sum_{i}{\left(\left|\sigma_{JL}^{k}\left(t_{i}\right)\right|-\left|{\hat{\sigma }}_{JL}^{k}\left(t_{i}\right)\right|\right)}^{2}}\;(19)$$

where *N* is the total number of timesteps in all trajectories.

The RMSD in Figure 2 shows that couplings computed from analytical second derivatives from a quadratic regression are always more accurate than couplings computed from numerical second derivatives. The difference between the two procedures is particularly remarkable for *δη* larger than 1 au ( Figure 2a). Nonetheless, the period Δ *T* over which the quadratic regression is done should be very short, smaller than 0.5 fs ( Figure 2b). This means that for dynamics with large timesteps, using the quadratic regression may not be advantageous. The parameters *δε* ( Figure 2c) and *δϖ* ( Figure 2d) play only a minor role in the BA couplings' accuracy.

**Figure 3.**
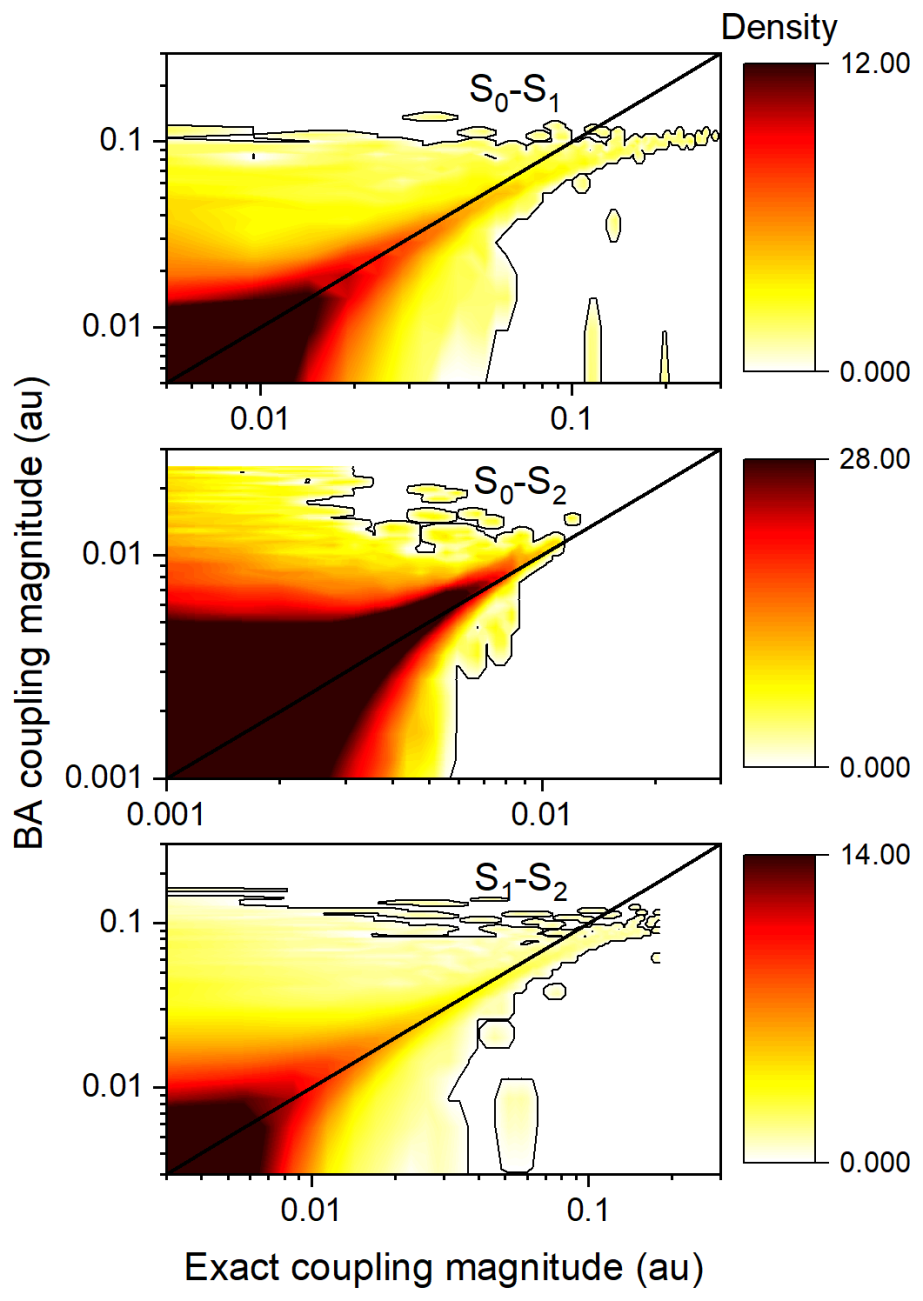
Density maps comparing the magnitudes of time-dependent Baeck-An (TD-BA) and exact nonadiabatic couplings for all points of each pair of states in the ethylene’s **h**-adjusted dataset. TD-BA; smoothed over Δ *T* = 0.4 fs, and with *δη* = 1 au, *δε* = 12 eV, and *δϖ* = 0.01 au. A low-level contour is shown in each graph to guide the eye. The maps were computed with 672,812 data points.

Density maps comparing the magnitudes of BA couplings to exact nonadiabatic couplings for all points of the **h**-adjusted dataset for ethylene are shown in Figure 3. As expected, most couplings are near zero. The largest couplings for each pair of states tend to fall on the diagonal, which implies a good agreement between TD-BA and the exact couplings. The order of magnitude is generally well represented, with the S _0_-S _2_ coupling magnitudes ten times smaller than those of the other two transitions. The TD-BA model tends to overestimate small-magnitude couplings, especially for the S _0_-S _2_ interactions. The model also shows some underestimation for large couplings in S _0_-S _1_ and S _1_-S _2_ interactions.

**Figure 4.**
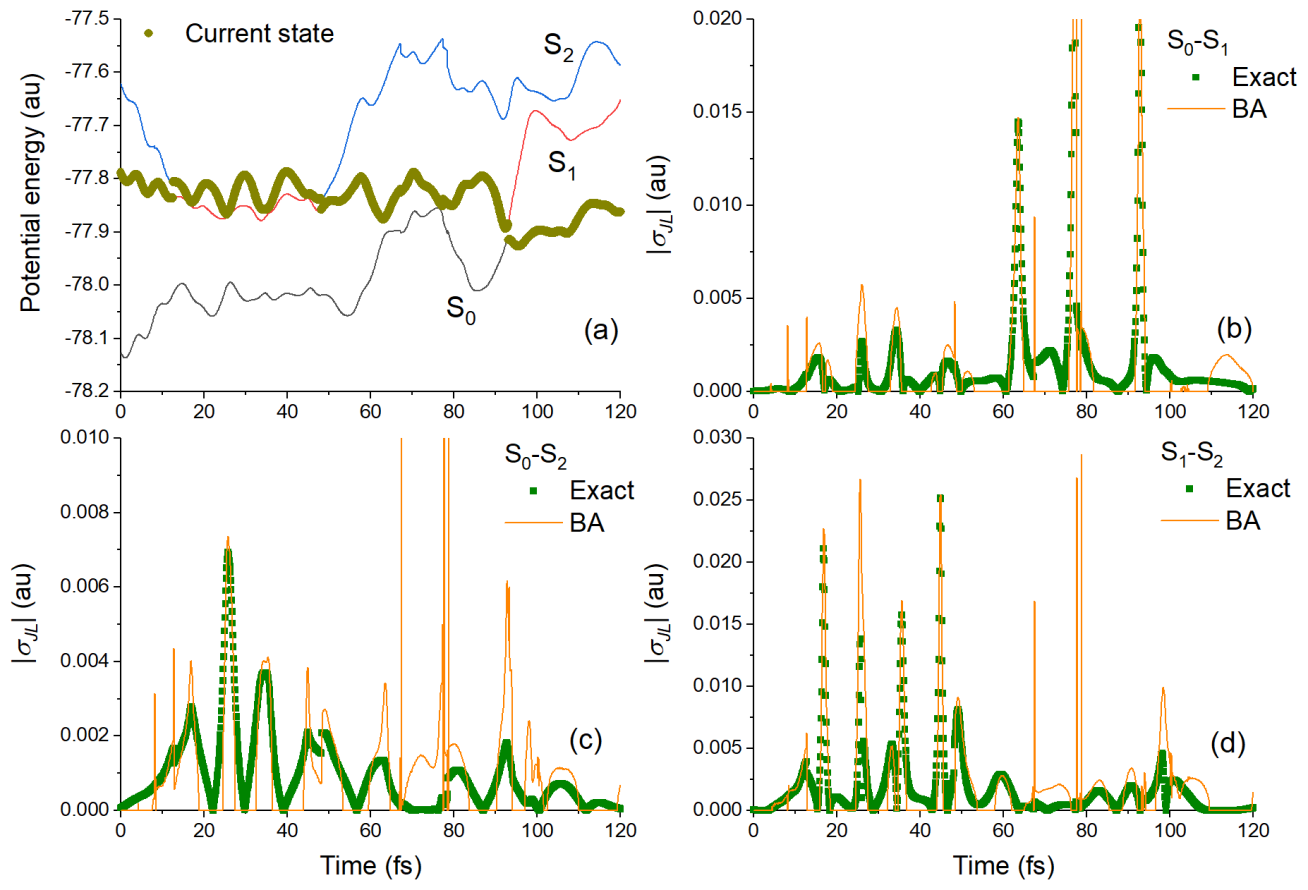
Benchmark tests on one trajectory (TRAJ1) from the ethylene’s h-adjusted dataset. ( **a**) Potential energies as a function of time. Exact and time-dependent Baeck-An (TD-BA) coupling magnitudes during this trajectory are shown in ( **b**) for S _0_-S _1_, ( **c**) for S _0_-S _2_, and ( **d**) for S _1_-S _2_.

Figure 4 shows results comparing TD-BA and exact couplings for a single trajectory of the **h**-adjusted dataset. In this case, after starting in S _1_, ethylene hops to S _2_ after 12.5 fs and stays there until it comes back to S _1_ at 48.1 fs. It finally returns to S _0_ at 93 fs ( Figure 4a). The TD-BA model delivers a semiquantitative agreement with the exact results for the coupling magnitudes between all three pairs of states ( Figure 4b, c, and d). The model correctly predicts the time where each peak occurs, as well as gives the right shape (intensity and width) of the coupling peaks. However, the TD-BA model shows spurious spikes that even the cleaning conditions discussed above could not get rid of.

### B. Potential use of TD-BA in TDDFT dynamics

Linear-response TDDFT is the workhorse of excited-state calculations of large molecular systems ^53^. Nevertheless, there are some critical obstacles to simulate nonadiabatic dynamics based on this method, especially when dealing with the internal conversion to the ground state. Because of its underlying approximations ^54^, TDDFT not only fails in describing the state crossing between the ground and the first excited state, but also predicts the wrong dimensionality of the branching space around the crossing ^55^. Such handicaps have not precluded cautious use of TDDFT for studying internal conversion to the ground state. Indeed, thanks to Send and Furche ^10^, S _1_/S _0_ nonadiabatic coupling vectors for TDDFT are now a common feature of several quantum chemistry software programs. Our own research protocol has been to run TDDFT dynamics until the energy S _1_/S _0_ gap drops below some threshold (about 0.1 eV), stop the trajectory propagation there, and assume that point to be the hopping point ^34^. Here, we were interested in testing whether the TD-BA model would allow going beyond this protocol, with actual hoppings to the ground state. To do so, we performed exploratory simulations with fulvene and thiophene.

The first challenge is to obtain an acceptable (TD) DFT description in regions close enough to the crossing region between the ground and excited state so that the hops between them can be computed. Therefore, we increased the size of the grid in the DFT calculations to the maximum value available in Turbomole 7.5 (grid size 7) as an attempt to avoid self-consistent field (SCF) convergence problems and negative excitation energies. Although this strategy successfully solved SCF convergence problems, our trajectories for fulvene were systematically terminated during the linear-response calculation of excitation energies due to singlet instabilities at small S _0_/S _1_ energy gaps. To further avoid singlet instabilities, we used the Tamm-Dancoff approximation (TDA) ^56^, as proposed by Tapavicza *et al.* ^57^ We computed 25 trajectories starting in the S _1_ state, of which 22 did not show any hopping between S _1_ and S _0_. In the remaining three trajectories, the hopping occurred at energy gaps of 0.41, 2.22, and 1.65 eV. This set of results suggests that the coupling between S _1 _and S _0 _is not well described for fulvene under the TDA approximation. Moreover, the S _1_/S _0_ energy gaps as a function of time were systematically too large (compared to those at CASSCF), indicating that the TDA description of the branching space was inadequate. Therefore, neither TDDFT nor TDA-DFT were suitable to be used for fulvene dynamics.

To test how system-dependent this failure was, we turned to thiophene. Surface hopping dynamics of thiophene with TDA was previously reported by Fazzi *et al.*, ^58^ who showed that TDA was able to describe the nonradiative relaxation process bringing thiophene to the S _1_/S _0_ crossing region. We computed 12 trajectories starting from the S _2_ state. From those, six hopped to the ground state with reasonably small energy gaps. Nevertheless, five prematurely ended due to singlet instability at the crossing region. We tried to avoid the singlet instability by decreasing the time step to 0.1 fs, hoping to have a hopping before reaching the instability point. However, the instability persisted.

Therefore, although the TD-BA model delivers an alternative way to compute the nonadiabatic couplings between the ground and excited states, its application to nonadiabatic dynamics at TDDFT level is discouraging since it still depends on the good description of the relevant states close to the crossing region. We expect similar negative results for dynamics based on algebraic diagrammatic construction to second-order (ADC(2)) ^59^, which tends to yield negative excitations near the S _1_/S _0_ crossing. Note, however, that TD-BA can still be helpful for both TDDFT and ADC(2) dynamics involving only internal conversion between excited states.

## VII. Dynamics results

### A. Ethylene

Ethylene dynamics has been extensively investigated with many theoretical approaches, including wavepacket dynamics ^60^, on-the-fly quasi-diabatic dynamics ^61^, multiple spawning ^62– 65^ multiconfigurational Ehrenfest ^66^, and surface hopping ^32, 67– 72^. The ultrafast nonadiabatic dynamics of ethylene is driven by torsional and pyramidalization modes until the molecule finds the S _1_/S _0_ crossing seam.

**Figure 5.**
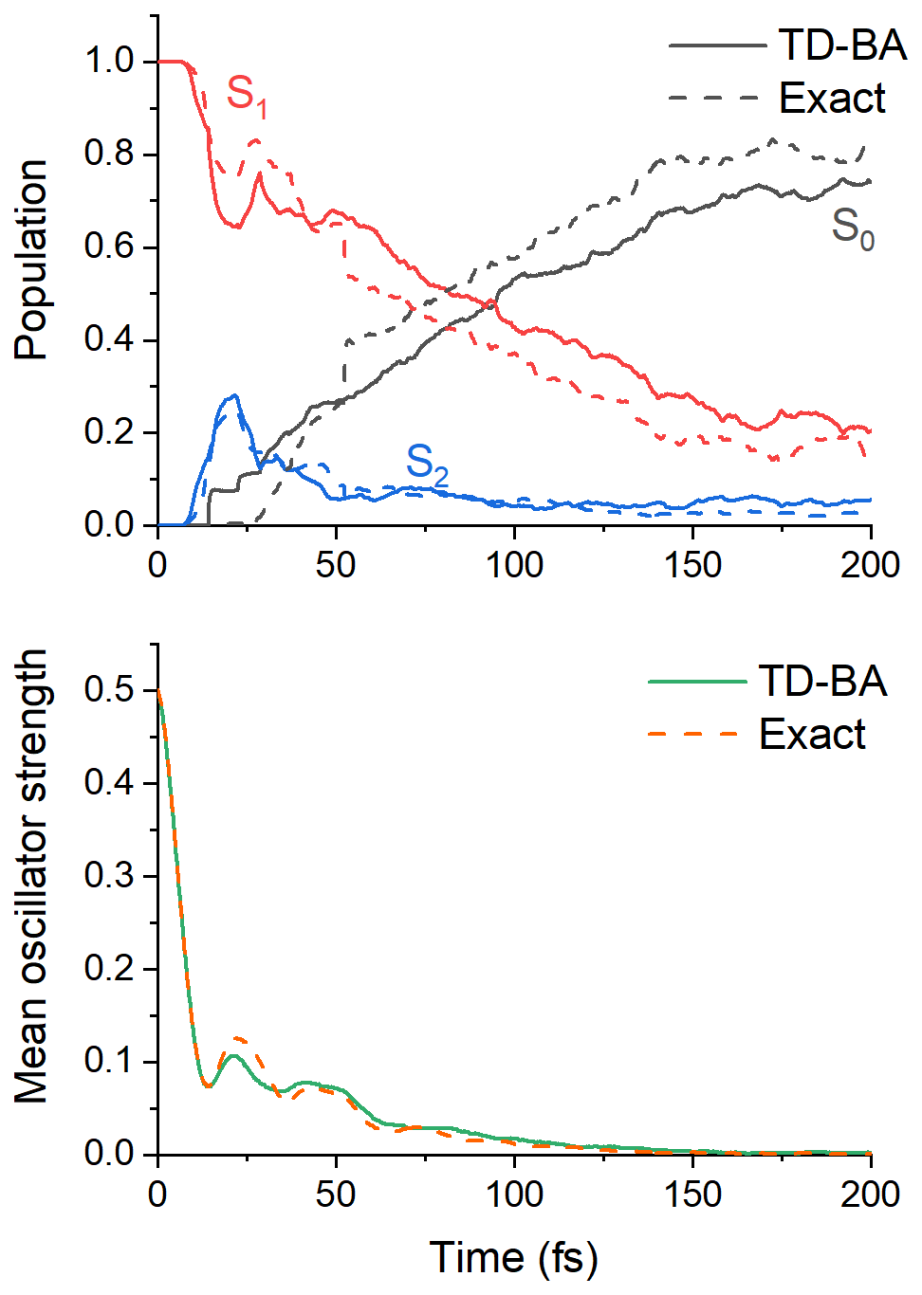
Time evolution of time-dependent Baeck-An (TD-BA) decoherence-corrected fewest switches surface hopping (DC-FSSH) dynamics of ethylene. (Top) mean adiabatic populations of S _0_, S _1_, and S _2_; (bottom) mean oscillator strength between the current and the ground states. "Exact" corresponds to DC-FSSH dynamics with exact couplings from the **h**-adjusted dataset.

The adiabatic-state population evolution based on TD-BA couplings is shown in Figure 5 (top). The S _1_ state is quickly depopulated toward S _0_ within 100 fs. In the first 20 fs, S _1_ also receives some of the population, but it quickly returns to the lower states. The S _1_ lifetime fitted with an exponential decay is 107 ± 9 fs, where the margin of error is computed for a 95% confidence interval.

Population evolutions with TD-BA and exact couplings are also compared in Figure 5 (top). The results are encouraging, delivering a semiquantitative agreement between the two datasets. The S _1_ lifetime in the **h**-adjusted dataset is 114 ± 10 fs, meaning that the results agree within the error bars. This level of variation is expected even within different sets of FSSH with exact couplings. For example, the lifetime of FSSH dynamics with exact couplings but adjusting velocities in the **p** direction is 120 ± 11 fs. Nevertheless, there are quantitative differences between TD-BA and the **h**-adjusted dataset. Although the TD-BA dynamics perfectly describes the S _2_ population evolution, the S _1_ and S _0_ evolutions bear some significant differences between the two datasets, with the S _1_ population after 50 fs always lower in the **h**-adjusted dataset.

The current state's mean oscillator strength is shown in Figure 5 (bottom) for TD-BA and the **h**-adjusted dataset. The time constant obtained from an exponential fitting is 14 ± 2 fs, in excellent agreement with the reference value, 15 ± 2 fs. TD-BA correctly describes the damped oscillations in ethylene dynamics, which have been previously predicted by wavepacket ^60^ and surface hopping ^67, 68^ simulations, and experimentally measured ^73, 74^. 

One of the most significant differences between the TD-BA and the **h**-adjusted datasets is the fraction of molecules still in the excited states after 200 fs ( Table 1). While the dynamics with exact couplings put this quantity at 9%, TD-BA dynamics has almost twice this amount, 16%. This disagreement is out of the margin of error of ±3%. Beyond this divergence, the ground-state-product' yields show much better agreement. At the end of the simulations, dynamics with TD-BA couplings predicts 38% of ground-state CH _2_CH _2_ structures, while dynamics with exact couplings, 39%. According to the TD-BA and reference sets, the expected amounts of ethylidene (CH _3_CH) are 14% and 18%, respectively. Single-H dissociation yields are 28% and 31%. Double-H dissociation yields are 1% and 3%. All these figures agree within their margin of errors.

**Table 1.** Mean value and error bars (95% confidence interval) of time constants, populations, and structural yields, computed for ethylene and fulvene dynamics. Ethylene’s results for the **h**-adjusted (reference) and **p**-adjusted datasets of Ref. 39 are shown as well. $\lambda_{x}^{h}$ and $\lambda_{x}^{p}$ are the overlap scores for each observable computed between time-dependent Baeck-An (TD-BA) and the respective dataset.

Observable	TD-BA	h dataset	( $\lambda_{x}^{h}$ )	p dataset	( $\lambda_{x}^{p}$ )
**Ethylene**					
Population time *τ _p_* (fs)	107±9	114±10	0.50	120±11	0.07
Osc. strength time *τ _o_* (fs)	14±2	15±2	0.73	17±2	0.02
S _2_, S _1_ CH _2_CH _2_ yield (%)	16±3	9±3	0.00	12±3	0.05
S _0_ CH _2_CH _2_ yield (%)	39±4	38±4	0.84	41±4	0.83
S _0_ CH _3_CH yield (%)	14±3	18±3	0.13	15±3	0.94
S _0_ C _2_H _3_ + H yield (%)	28±4	31±4	0.60	27±4	0.83
S _0_ C _2_H _2_ + 2H yield (%)	1±1	3±2	0.22	3±1	0.01
**Fulvene**					
1 ^st^ decay time *τ _1_* (fs)	11±1	9±1	0.00	9±1	0.00
S _1_ population at 20 fs (%)	46±7	34±6	0.00	42±7	0.66
S _1_ population at 60 fs (%)	12±5	3±3	0.00	6±3	0.02
Planar S _1_→S _0_ hop (%)	85±5	87±5	0.81	84±5	0.93
Tw-Stretched S _1_→S _0_ hop (%)	5±3	6±3	0.86	8±4	0.38
Tw-Shrunk S _1_→S _0_ hop (%)	9±4	7±4	0.73	8±4	0.91

The hopping counting and the mean energy gap at the hopping time for all transitions are shown in Table 2 for the three datasets. Concerning the mean gap, TD-BA is in excellent agreement with the other two sets for all transitions excepting S _0_→S _2_. This discrepancy is not statistically relevant given that there are only a few of such rare transitions in each set. The number of hoppings between S _1_ and S _0_ (both directions) also agrees well between TD-BA and the other two sets. Nevertheless, the number of hoppings between S _1_ and S _2_ (both directions) is twice as large in TD-BA as in the other sets. With exact couplings, 43% of trajectories populated S _2_. These trajectories returned to S _1_ afterward and, in general, remained there until they hopped to S _0_. With TD-BA couplings, the fraction of trajectories populating S _2_ was higher, 63%. These trajectories usually hopped forth and back between S _1_ ad S _2_ twice before converting to S _0_. 

**Table 2.** Number of hoppings (count) and hopping energy-gap absolute mean value and standard deviation for the three sets of trajectories of ethylene and fulvene.

	TD-BA set			h set			p set		
	Count	Mean (eV)	St. Dev. (eV)	Count	Mean (eV)	St. Dev. (eV)	Count	Mean (eV)	St. Dev. (eV)
**Ethylene**									
S _2_→S _1_	520	0.35	0.22	259	0.39	0.28	252	0.43	0.32
S _2_→S _0_	38	4.63	0.42	18	4.93	0.47	20	4.91	0.97
S _1_→S _2_	563	0.61	0.45	281	0.63	0.43	285	0.62	0.42
S _1_→S _0_	546	0.51	0.63	618	0.65	0.88	613	0.61	0.85
S _0_→S _2_	4	3.91	0.54	2	2.12	0.29	6	1.88	0.19
S _0_→S _1_	160	0.71	0.81	181	0.76	0.86	187	0.70	0.82
**Fulvene**									
S _1_→S _0_	273	0.25	0.27	273	0.28	0.28	278	0.25	0.25
S _0_→S _1_	92	0.57	0.56	82	0.56	0.40	91	0.47	0.32

**Figure 6.**
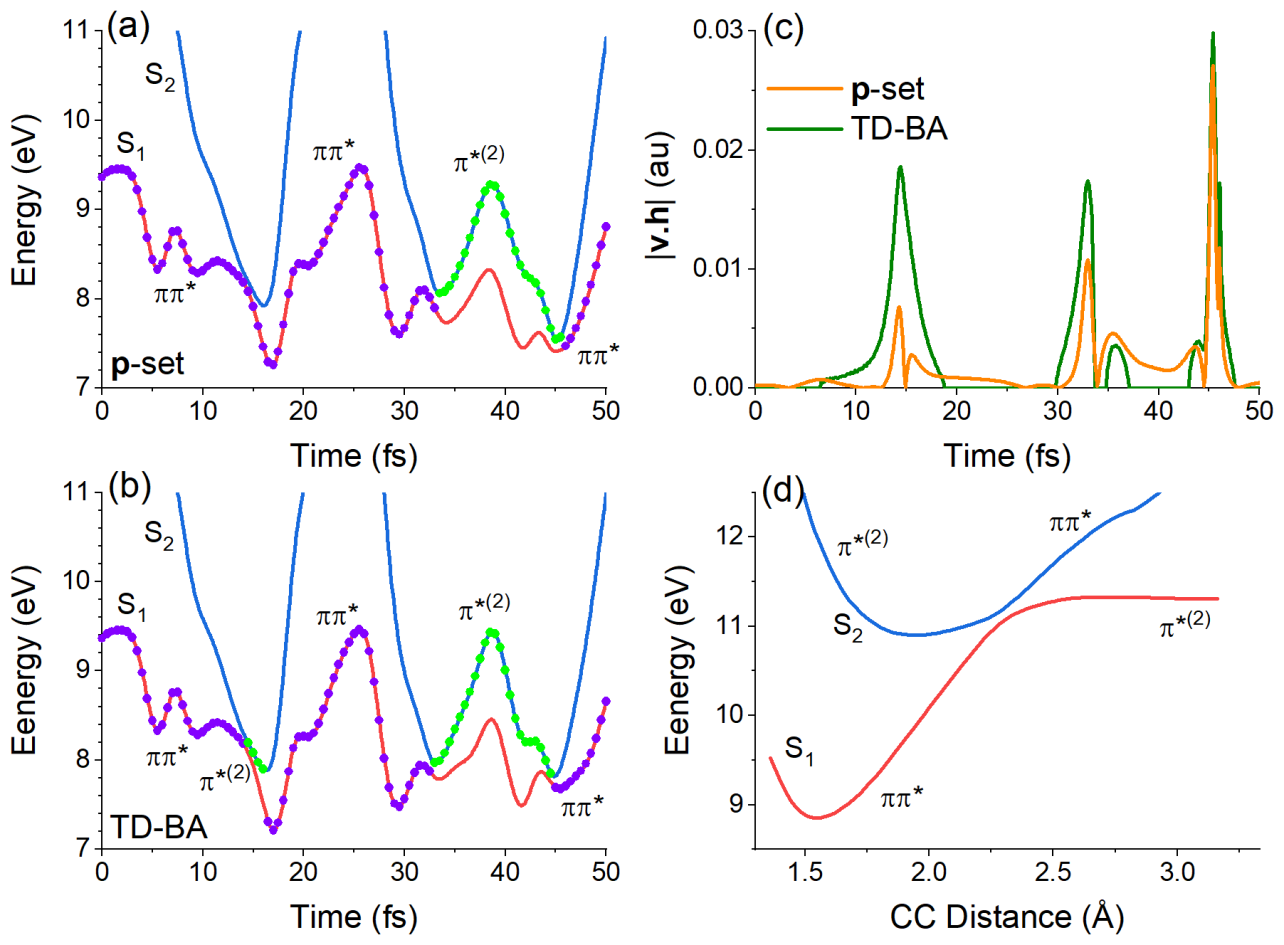
Characterization of the first 50 fs of a single trajectory of ethylene. Potential energies of the S _1_ and S _2_ states as a function of time with ( **a**) exact **p**-adjusted dataset and ( **b**) time-dependent Baeck-An (TD-BA) couplings. The dots indicate the current state every 0.5 fs. Violet dots indicate ππ* diabatic character, and green dots indicate π* ^(2)^ character. The absolute value of the exact and TD-BA **v**. **h** projection computed for the reference trajectory is shown in ( **c**). The potential energy profile of S _1_ and S _2_ along the CC distance, keeping the other coordinates at the S _0_ minimum, is given in ( **d**).

We can understand the excess of S _1_-S _2_ hoppings in the TD-BA approach by analyzing a single trajectory computed with exact and TD-BA couplings, as illustrated in Figure 6. The comparison is made to a **p**-adjusted dataset trajectory, limiting the differences to the coupling itself, without the influence of the velocity adjustment after hopping.

In the initial stage of the S _1_ dynamics, the CC stretching strongly stabilizes the zwitterionic state S _2_ (π* ^(2)^). As shown in Figure 6a, the S _1_ and S _2_ states approach each other every approximately 15 fs. S _2_ destabilizes again when the CC bond shrinks due to the oscillatory motion. This initial oscillation of the S _1_-S _2_ gap is a common feature of all trajectories. When dynamics is propagated with TD-BA couplings, the hopping probability from S _1_ (ππ*) to S _2_ (π* ^(2)^) at the stretched structure is larger than when propagating with exact couplings. Thus, the TD-BA trajectories hop to S _2_ about twice more often than the reference trajectories.

The reason for the larger probability is connected to the surface topography. With the exact coupling, the projection **h** on **v** is modulated by the velocity magnitude. When the CC stretching is maximum, the velocity in this internal coordinate is null, and **v**. **h** is null as well, causing the zero at 15 fs that we can see in the orange curve ( **p**-adjusted dataset) in Figure 6c. TD-BA coupling fails to capture this dependence on | **v**|, yielding a larger coupling peak at the same time. Nevertheless, this discrepancy in the probability does not play a significant role in the dynamics — at least not for ethylene. After populating S _2_, the molecule quickly returns to S _1_ within 5 fs or less, and the population evolution is not affected, as we have seen in Figure 5.

Table 1 gives the overlap score for all time constants and structural yields discussed above. When the TD-BA dataset is compared to the **h**-adjusted dataset, the scores ( $\lambda_{x}^{h}$ ) are excellent for the S _0_ CH _2_CH _2_ yield (0.8) and oscillator strength time constant (0.7). For the population time constant (0.5) and single-H dissociation (0.6), the scores are fine. They are poor for the less populated channels, ethylidene (0.1) and double-H dissociation (0.2). The results do not agree at all for excited-state CH _2_CH _2_ (0.0).

When the TD-BA dataset is compared to the **p**-adjusted dataset $\lambda_{x}^{p}$ , the lifetime agreement deteriorates (0.07 score for population and 0.02 for oscillator strength), but most of the structural figures improve ( Table 1). There is an almost perfect agreement for ground-state CH _2_CH _2_, CH _3_CH, and single H dissociation yields between the two sets, with overlap scores above 0.8. This means that part of the divergence to the **h**-adjusted dataset is caused not by the TD-BA couplings but by the **p**-adjustment of the velocities, which directly impacts the fragmentation process.

The statistical analysis in this paper follows the methods and protocol defined proposed in Ref. 39 Thus, the current results can be directly compared to those previously published datasets. Table 3 compiles a list of mean overlap scores Λ ^(1,2)^, comparing different FSSH setups to the **h**-adjusted reference dataset. The idea is that the implementation of new methods and testing of different features can always be analyzed in the same way to build a hierarchical comparison of procedures. We see that TD-BA yields Λ ^(1,2)^ of 0.43 at the same level as that of the **p**-adjusted dataset (0.41). This is equivalent to say that the results of ethylene DC-FSSH dynamics with TD-BA couplings and with exact couplings adjusting the velocity in the **p** direction are statistically equivalent for 500 trajectories and 95% confidence interval. Nevertheless, this statement does not mean that TD-BA results should be accepted without questioning. The average overlap score varies between 0 (complete disagreement with reference data) and 1 (perfect agreement). An average overlap score of 0.43 means that significant differences may be expected in comparison to the reference data. However, these differences are not worse than when we use exact couplings and do velocity adjustment in the **p** direction. As we discussed in the previous paper ^39^, if we reduce the number of trajectories in the ensemble, the margins of error grow, and all overlap scores improve. For a small but typical ensemble of 100 trajectories, the mean overlap score for TD-BA dynamics is about 0.7, which is an acceptable agreement with the reference data. Therefore, TD-BA dynamics seems to be a promising approach for exploratory dynamics with few trajectories.

**Table 3.** Mean overlap score Λ ^(1,2)^ computed for different fewest switches surface hopping (FSSH) setups. Ethylene: 500 trajectories in each dataset and 95% confidence interval averaged over seven observables. Fulvene: 200 trajectories in each dataset and 95% confidence interval averaged over six observables. The **h**- and **p**-adjusted datasets of ethylene are from Ref. 39.

Dataset	Λ ^(1,2)^	
	Ethylene	Fulvene
**h**-adjusted (reference)	1	1
**p**-adjusted	0.41	0.59
TD-BA	0.43	0.40

### B. Fulvene

Fulvene nonadiabatic dynamics has been discussed before based on different simulation approaches, including wavepacket propagation in reduced-dimensionality ^75– 79^, full-dimensionality direct-surface wavepacket propagation ^80, 81^, multiple spawning ^32^, and surface hopping ^32, 82^. Fulvene has an extended S _1_/S _0_ crossing seam from planar to 90° twisted structures ^36, 82, 83^. The central feature of its photophysics is the ultrafast sub-100 fs decay to the ground state, with periodic returns to S _1_ and internal conversion spread over the entire crossing seam.

For the analysis that follows, it is helpful to define the mean torsional angle as the average of the absolute values of the four dihedral angles around the C–CH _2_ bond, which in degrees is:

$$\phi_{{\text{C-CH}}_{\text{2}}}=\frac{1}{4}\left[\left|\phi_{\text{CC-CH}}^{cis_{1}}\right|+\left|\phi_{\text{CC-CH}}^{cis_{2}}\right|+\left|180^{{}^{\circ}}-\phi_{\text{CC-CH}}^{trans_{1}}\right|+\left|180^{\circ }-\phi_{\text{CC-CH}}^{trans_{2}}\right|\right]\;(19)$$

**Figure 7.**
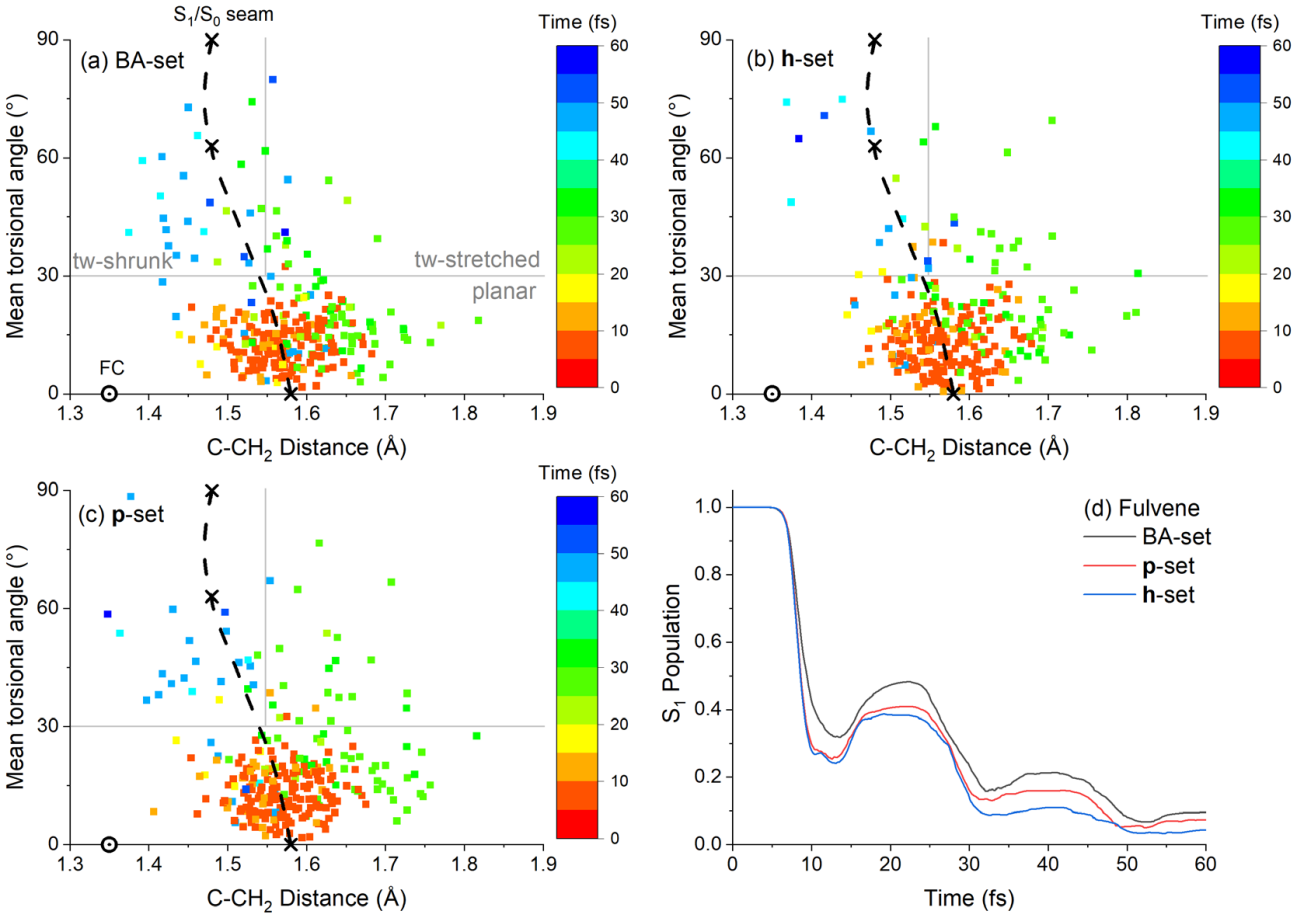
Characterization of fulvene dynamics. C–CH _2_ mean dihedral angle and the CC distance at the S _1_→S _0_ hopping for the ( **a**) time-dependent Baeck-An (TD-BA), ( **b**) **h**-adjusted, and ( **c**) **p**-adjusted datasets. The colors indicate the hopping time. The dotted circle shows the Franck-Condon (FC) position, and the crosses show the positions of the planar conical intersection (0°), the minimum on the crossing seam (63°), and the twisted conical intersection (90°). The dashed curve indicates the minimum on the crossing seam optimized with constrained angular values. The vertical and horizontal lines split the space into three regions, Planar ( *ϕ*_C–CH _2__ < 30°), Twisted-stretched ( *ϕ*_C–CH _2__ ≥ 30°, *d*_C–CH _2__ ≥ 1.55 Å), and Twisted-shrunk ( *ϕ*_C–CH _2__ ≥ 30°, *d*_C–CH _2__ < 1.55 Å). Panel ( **d**) shows the S _1_ population as a function of time for the three datasets.

To compare the results of different datasets, we have defined three regions in the C–CH _2_ torsion and distance space. The first region (Planar) includes near planar structures with a mean torsional angle smaller than 30°. The second region (Twisted-stretched) includes twisted structures (≥ 30°) with a C–CH _2_ distance larger than 1.55 Å. The third region (Twisted-shrunk) includes twisted structures with C–CH _2_ distances smaller than 1.55 Å. The count of S _1_→S _0_ hoppings in each region is given in Table 1. These three regions are indicated in Figure 7a-c.

The three datasets show that same general behavior. After the excitation, fulvene returns to the ground state within 10 fs, with S _1_ repopulations (recurrences) at 20, 40, and 60 fs ( Figure 7d). Internal conversion to S _0_ can occur at different points of the crossing seam. As shown in Figure 7 (a-c), the first wave of S _1_→S _0_ hoppings occurs in the Planar region, in the first 20 fs after excitation. In the **h**-adjusted dataset ( Figure 7b), the mean value of this angle is 13° (±7°), and the mean C–CH _2_ distance is 1.58 Å (±0.06 Å). For comparison, the crossing seam has a maximum at 0° and 1.58 Å, implying that the first wave of S _1_→S _0_ hoppings occurs near this maximum. The second S _1_→S _0_ hopping wave happens between 20 and 40 fs at stretched C-CH _2_ distances (> 1.55 Å). In this second wave, the structure may be either planar or twisted. A third S _1_→S _0_ hopping wave happens between 40 and 60 fs, mostly at the Twisted-shrunk region. This third wave occurs near the minimum of the crossing seam, located at 63° and 1.48 Å.

To proceed with the statistical analysis, including the calculation of overlap scores, the S _1_ population error bar (95% confidence interval) was estimated with bootstrap through 10,000 repetitions of the 200 trajectories of each dataset.

**Figure 8.**
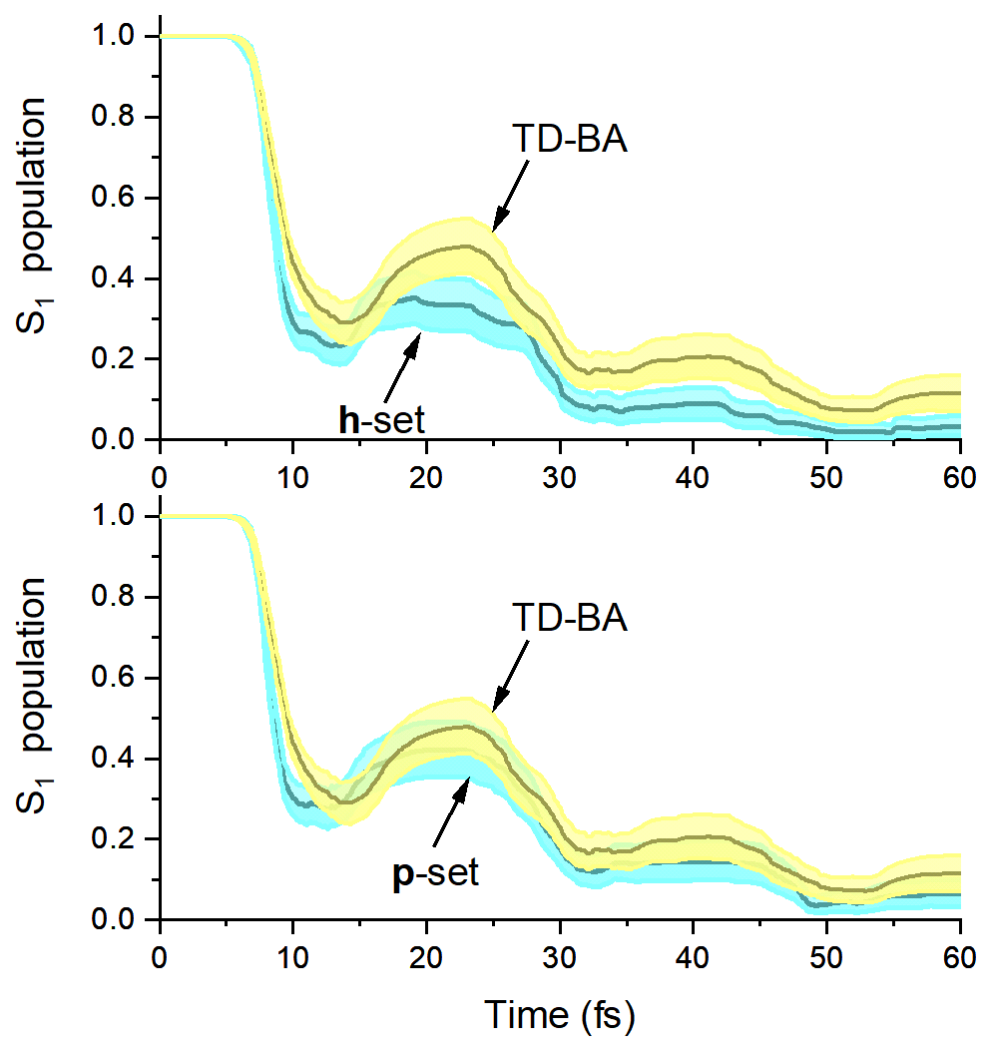
Fulvene S _1_ population. Top: time-dependent Baeck-An (TD-BA) and **h**-adjusted dataset; bottom: TD-BA and **p**-adjusted dataset. In each case, the shaded area indicates the 95% confidence interval sampled with bootstrap through 10,000 repetitions of the 200 trajectories of each dataset.

The overlap scores are above 0.7 for the number of S _1_→S _0_ hoppings in each of the three regions of the torsion × distance space ( Table 1). It means that the TD-BA model quantitatively agrees with both **h**- and **p**-adjusted datasets for predicting these observables. The only exception is the divergence in the number of Twisted-stretched hoppings between TD-BA and the **p**-adjusted dataset (0.38 overlap score). The overlap scores for the time and population observables do not agree so well, with values zero or near zero. Nevertheless, this divergence in the population evolution is not critical for the validity of the TD-BA model. As shown in Figure 8, the TD-BA dynamics follow the same qualitative trends in the **h-** and **p**-adjusted datasets. However, the initial TD-BA evolution is slightly slower, 11 ± 1 fs against 9 ± 1 fs of other datasets. Moreover, the TD-BA S _1_ population tends to be higher, especially when compared to the **h**-adjusted dataset. When considering all six observables, the mean overlap score Λ ^(1,2)^ between TD-BA and the **h**-adjusted reference dataset is 0.40 ( Table 3), not much worse than the 0.59 obtained in the comparison between the **p**-adjusted dataset and the **h**-adjusted dataset.

As a final remark, in Ref. 32, Ibele and Curchod reported surface hopping dynamics results for fulvene, also computed at CAS(6,6) level. Their S _1_ population at 20 fs computed with **p**-adjusted DC-FSSH was, like ours, near 0.4 (see Figure 4 of that paper). Nevertheless, with **h**-adjusted DC-FSSH, this observable was reduced to about 0.2, which disagrees with our results. Although we cannot provide a definitive explanation for the divergence, it may be that their trajectory ensemble composed of 18 initial conditions repeated 10 times each is too biased to deliver a reliable population.

## VIII. Conclusions

In Ref. 27, Baeck and An showed that the first-order nonadiabatic coupling vector could be approximated by a 1D model, depending only on the energy gap and the second derivative of this gap with respect to the coupling coordinate (see Equation (1)). In this paper, the BA approximation is recast on the time domain, giving rise to the TD-BA approximation ( Equation (12)), which can be applied to multidimensional systems. The TD-BA couplings are equivalent to a coupling projection on the nuclear velocities, making them particularly suited to be employed in DC-FSSH simulations. TD-BA couplings immediately enable nonadiabatic dynamics with any electronic method that can provide excitation energies and energy gradients. 

We have used the methodological protocols proposed in Ref. 39 to evaluate dynamics based on TD-BA couplings. Extensive simulations for ethylene and fulvene at MCSCF level showed that TD-BA DC-FSSH provides a correct qualitative picture of the dynamics and delivered results in semiquantitative agreement with the reference datasets computed with exact nonadiabatic couplings. The results' quality is statistically not much worse than performing DC-FSSH adjusting velocities after hopping in the momentum direction. Nevertheless, the model fails to adequately describe the nonadiabatic dynamics in regions with strong nonadiabatic couplings but small velocities (which are themselves challenging regions for surface hopping in general ^84^).

In our implementation of the DC-FSSH with TD-BA couplings, we tested two ways of computing second time derivatives of the energy gaps and proposed three conditions to be satisfied to reduce numerical instabilities in the calculations. Although this initial modeling has delivered adequate results, there is a margin for further improvements, especially in detecting artifacts caused by small discontinuities in the potential energy surface.

We have also tested the TD-BA DC-FSSH with TDDFT to check whether this method would allow predicting hoppings to the ground state. In this case, however, the answer was negative due to singlet instabilities. This problem is not restricted to TD-BA and should happen in TDDFT dynamics based on exact couplings too. Dynamics based on TD-BA with TDDFT may still help describe internal conversion between excited states. 

Given the uncertainties introduced by the TD-BA approximation, DC-FSSH dynamics with these couplings should be restricted to exploratory dynamics, where high accuracy is not a strong requirement. In this work, we have tested the model for the nonadiabatic dynamics of ethylene and fulvene. Although together these two molecules are representative of many typical excited-state cases, dynamics with TD-BA couplings should be thoroughly tested before being applied to other types of topography and densities, like in superexchange ^85^, spin-exchange ^86^, solvent-solute charge-transfer ^87^, three-states crossing ^88^, and dissociative ^89^ internal conversion.

## Data availability

### Underlying data

Figshare: Fulvene DC-FSSH. https://doi.org/10.6084/m9.figshare.14446998.v1 ^44^.

This project contains the following underlying data:

- All Newton-X input and output files for dynamics of fulvene (text format) with TD-BA, **h**-adjusted velocities, and **p**-adjusted velocities.

Figshare: TD-BA DC-FSSH Dynamics. https://doi.org/10.6084/m9.figshare.14010311.v1 ^45^.

This project contains the following underlying data:

- All Newton-X input and output files for dynamics of ethylene (text format) with TD-BA.

Figshare: ethylene-dyn-2021.tgz. https://doi.org/10.6084/m9.figshare.13522856.v2 ^46^.

This project contains the following underlying data:

- All Newton-X input and output files for dynamics of ethylene (text format) with **h**-adjusted velocities and **p**-adjusted velocities.

Data are available under the terms of the Creative Commons Attribution 4.0 International license (CC-BY 4.0).
