# Supplementary material for: Fewest switches surface hopping with Baeck-An couplings
Source: Open Res Eur. 2022 Mar 9;1:49. Originally published 2021 May 17. [Version 2] doi: 10.12688/openreseurope.13624.2 (PMC10446015; doi:10.12688/openreseurope.13624.2)
Supplement: Peer review history

# Peer review: Reviewer response for version 1

**DOI:** 10.21956/openreseurope.14695.r26914

**Published:** 2021-06-02

**Authors:** Lluís Blancafort

**Affiliations:** 1 Department of Chemistry and Institute of Computational Chemistry and Catalysis. Faculty of Sciences, University of Girona, Girona, Spain

This manuscript introduces a new approximation to calculate non-adiabatic couplings for trajectory surface hopping, based on an expression introduced previously by Baeck and An. The development of new schemes for coupling calculation is important to make excited-state simulations more efficient. The authors provide a detailed description of their new approximation that ensures that it can be fully reproduced. The same can be said about the applications (fulvene and ethylene excited state trajectories). Therefore, the work is fully suitable for indexing in Open Research Europe.

 As a minor revision, I would ask the authors to comment on the validity of the assumption that the only non-null energy Hessian component is the one in the Q direction, where Q is the coupling coordinate (see Section III). This assumption seems key to extend the BA's approach to multidimensional systems. However in polyatomic molecules the opposite often occurs, i.e. the S0 and S1 surface have different curvature along several vibrational coordinates. This has been studied in detail e.g. for fulvene ^1^, and large differences in the S0 and S1 Hessian are found along symmetry-preserving coordinates where the coupling is zero. Will the new approach not overestimate the coupling in such cases?

Is the rationale for developing the new method (or application) clearly explained?

Yes

Is the description of the method technically sound?

Yes

Are the conclusions about the method and its performance adequately supported by the findings presented in the article?

Yes

If any results are presented, are all the source data underlying the results available to ensure full reproducibility?

Yes

Are sufficient details provided to allow replication of the method development and its use by others?

Yes

Reviewer Expertise:

Computational chemistry.

I confirm that I have read this submission and believe that I have an appropriate level of expertise to confirm that it is of an acceptable scientific standard.

## References

[1] : The curvature of the conical intersection seam: an approximate second-order analysis. *J Chem Phys* .2004;121(23) : 10.1063/1.1813436 11562-71 10.1063/1.1813436 15634121

# Peer review: Reviewer response for version 1

**DOI:** 10.21956/openreseurope.14695.r26913

**Published:** 2021-06-01

**Authors:** Julia Westermayr

**Affiliations:** 1 Computational Surface Chemistry Group, Department of Chemistry, University of Warwick, Coventry, UK

The article “Fewest switches surface hopping with Baeck-An couplings” by Mariana T. do Casal *et al. *represents a detailed analysis of the Baeck-An couplings within a time-dependent approximation (TD-BA) that can be used in fewest-switches surface hopping molecular dynamics simulations. This approach is especially powerful as it can enable surface hopping dynamics using only predicted energies and forces. Their analysis is based on the ultrafast dynamics of ethylene and fulvene at MCSCF level, two molecules recently considered by Ibele *et al.* as molecular Tully models for benchmarking different nonadiabatic dynamics methods. The authors compare their results with nonadiabatic dynamics with “exact” nonadiabatic coupling vectors, i.e., computed with the reference method, in combination with velocities adjusted in momentum and nonadiabatic coupling direction. Their results show that the TD-BA approximation leads to qualitatively correct trends in the dynamics and a semiquantitative agreement with respect to the reference dynamics. The authors further tested the method for fulvene and thiophene at the TDDFT level of theory. However, the use of the TD-BA model is restricted to excited state hoppings. Hoppings from an excited state to the ground state remain problematic, but this problem is also experienced in surface hopping dynamics with “exact” nonadiabatic coupling vectors. Their work clearly demonstrates that the TD-BA approximation can become a comparative method for studying the photodynamics of molecular systems when an accurate reference method is available and clearly highlights the discrepancies and uncertainties of the different flavours of surface hopping dynamics.

 Overall, the authors have presented their manuscript in a clearly structured manner and I enjoyed reading the article. The manuscript is well written, it is easy to follow how conclusions are drawn and explanations are provided when needed. I especially think that the authors have done a fantastic job of laying out the technical details in Section VI A. This section explains how to obtain the second time derivative of the energy gap and what approaches are tested to address the discontinuities in the potential energy landscapes introduced by the reference method.

 I have a few minor suggestions that can further improve the manuscript:

1. To analyse the differences between the methods, the authors use an (average) overlap score that was also used in Ref. 39, in which the corresponding author compared the surface hopping dynamics of ethylene with different types of velocity adjustment. Twice in the text, the reader is referred to Ref. 39. I believe that a brief description of the method (in somewhat more detail than in the second paragraph on page 6) would benefit readers.
2. The authors describe their efforts to simulate the dynamics of fulvene using TDDFT and TDA-DFT. Due to problems in the reference simulations, the authors tested their method for the surface hopping dynamics of thiophene. Besides this reason for studying thiophene, it would be interesting to know what other reasons led to the choice of thiophene. A brief description of its dynamics and why it is particularly suitable for the purpose of this study would be beneficial.
3. Ibele *et al.* (ref. 32) proposed three Tully models, i.e., ethylene, fulvene, and DMABN, to benchmark surface hopping methods. Is there any reason why the authors have chosen ethylene and fulvene and did not consider DMABN? I do not expect the authors to perform their analysis for the third molecule, but I believe that a sentence or two in which the authors explain their choice might be helpful. I would also appreciate it if the authors could comment on the expected performance of the TD-BA approximation in studying the dynamics of DMABN.
4. Page 6, first paragraph: I believe there is a typo and " *εω*" should read " *δω*".
5. Page 8, Fig. 4 (d): The authors state that “the TD-BA model shows spurious spikes that even the cleaning conditions discussed above could not get rid of.” However, looking at Fig. 4(a) suggests that the spikes between 60 and 80 fs could be eliminated with a small enough value of *δ* *ε* as the energy gap between S1 and S2 seems to be in the range of 5 eV.
6. If the authors wish, they could slightly improve Fig. 7, as I think this graph could benefit from molecular structures of fulvene.

Besides these minor comments, I do not have any further suggestions and believe that the article meets the criteria for indexing in Open Research Europe.

Is the rationale for developing the new method (or application) clearly explained?

Yes

Is the description of the method technically sound?

Yes

Are the conclusions about the method and its performance adequately supported by the findings presented in the article?

Yes

If any results are presented, are all the source data underlying the results available to ensure full reproducibility?

Yes

Are sufficient details provided to allow replication of the method development and its use by others?

Yes

Reviewer Expertise:

Nonadiabatic molecular dynamics, surface hopping, nonadiabatic coupling approximation, machine learning.

I confirm that I have read this submission and believe that I have an appropriate level of expertise to confirm that it is of an acceptable scientific standard.

# Response

**Published:** 2022-02-28

**Authors:** Mario Barbatti

**Affiliations:** 178807, France

We thank the reviewer for the detailed analysis of our work. We provide a point-by-point answer below. We are also submitting a new version of the manuscript to reflect these answers. (Reviewer comments in itlaics)

*To analyse the differences between the methods, the authors use an (average) overlap score that was also used in Ref. 39, in which the corresponding author compared the surface hopping dynamics of ethylene with different types of velocity adjustment. Twice in the text, the reader is referred to Ref. 39. I believe that a brief description of the method (in somewhat more detail than in the second paragraph on page 6) would benefit readers.*

**Response:** We extended the explanation of this quantity in the last paragraph of Section V.

*The authors describe their efforts to simulate the dynamics of fulvene using TDDFT and TDA-DFT. Due to problems in the reference simulations, the authors tested their method for the surface hopping dynamics of thiophene. Besides this reason for studying thiophene, it would be interesting to know what other reasons led to the choice of thiophene. A brief description of its dynamics and why it is particularly suitable for the purpose of this study would be beneficial.*

**Response:** The main reason we chose thiophene is that we had studied this molecule with TDDFT before and got satisfactory results. This is explained in Section VI-b: “Surface hopping dynamics of thiophene with TDA was previously reported by Fazzi *et al*., who showed that TDA was able to describe the nonradiative relaxation process bringing thiophene to the S _1_/S _0_ crossing region.”

*Ibele et al. (ref. 32) proposed three Tully models, i.e., ethylene, fulvene, and DMABN, to benchmark surface hopping methods. Is there any reason why the authors have chosen ethylene and fulvene and did not consider DMABN? I do not expect the authors to perform their analysis for the third molecule, but I believe that a sentence or two in which the authors explain their choice might be helpful. I would also appreciate it if the authors could comment on the expected performance of the TD-BA approximation in studying the dynamics of DMABN.*

**Response:** As explained in the paper, we have chosen fulvene because it “poses the challenge of recurrences at an extended crossing seam with both peaked and sloped conical intersections.” At this moment, we do not have enough information to tell how TD-BA would perform for DMABN. Nevertheless, we have plans to extend benchmark simulations to include this molecule.

*Page 6, first paragraph: I believe there is a typo and "εω" should read "δω".*

**Response:** The typo was fixed.

*Page 8, Fig. 4 (d): The authors state that “the TD-BA model shows spurious spikes that even the cleaning conditions discussed above could not get rid of.” However, looking at Fig. 4(a) suggests that the spikes between 60 and 80 fs could be eliminated with a small enough value of δε as the energy gap between S1 and S2 seems to be in the range of 5 eV.*

**Response:** Our tests did not show significant quality improvement changing de. Nevertheless, it is possible that the set of parameters that we have used is not optimal. One of the plans for future developments of the method is improve the parameterization procedure. We have noticed that there was a mistake in the scale of Figure 2a, and the values of dh were multiplied by a factor 10. The figure was corrected in the revision.

*If the authors wish, they could slightly improve Fig. 7, as I think this graph could benefit from molecular structures of fulvene. The shrunk/stretched C-CH _2_ distance is not a feature easily distinguishable by visual inspection*.

**Response:** We are afraid that adding these structures would overcrowd the figure and not aid the reader.

# Peer review: Reviewer response for version 1

**DOI:** 10.21956/openreseurope.14695.r26916

**Published:** 2021-05-25

**Authors:** Sebastian Fernadez-Alberti

**Affiliations:** 1 Universidad Nacional de Quilmes, Bernal, Argentina

The authors statistically analyzed the different approximations by which the Baeck-An couplings can be implemented is FSSH algorithms. They have tested the method using ethylene and fulvene molecules. They provide approximations given in eq. 12 and also the calculation of FSSH probability(eq. 13). Additional TD-BA DC-FSSH calculations were also done with linear-response time-dependent density functional (TDDFT). Besides, the authors explain how the second time derivative in Equation (12) can be computed numerically or analytically using eq. 15-17. The article property explains the different approximation, achieved accuracies and benchmark tests. They proved that TD-BA couplings are equivalent to a coupling projection on the nuclear velocities, making them particularly suited to be employed in DC-FSSH simulations. Extensive simulations for ethylene and fulvene at MCSCF level showed that TD-BA combined with DC-FSSH provide a correct qualitative picture of the dynamics and delivered results in semiquantitative agreement with the reference datasets computed with exact nonadiabatic couplings. The results’ quality is statistically not much worse than performing DC-FSSH adjusting velocities after hopping in the momentum direction. Nevertheless, the model fails to adequately describe the nonadiabatic dynamics in regions with strong nonadiabatic couplings but small velocities. Finally, the authors test two different ways of computing second time derivatives of the energy gaps and proposed three conditions to be satisfied to reduce numerical instabilities in the calculations.

 Although this initial modeling has delivered adequate results, the authors claim that there is a margin for further improvements, especially in detecting artifacts caused by small discontinuities in the potential energy surface. The article is well written. I encourage the authors to describe the further improvements they have made and, in case they have performed preliminary tests, perhaps they can describe their findings and, in this way, help other authors future attempts of implementations. Despite that I think the article merits indexing at Open Research Europe.

Is the rationale for developing the new method (or application) clearly explained?

Yes

Is the description of the method technically sound?

Yes

Are the conclusions about the method and its performance adequately supported by the findings presented in the article?

Yes

If any results are presented, are all the source data underlying the results available to ensure full reproducibility?

Yes

Are sufficient details provided to allow replication of the method development and its use by others?

Yes

Reviewer Expertise:

NA

I confirm that I have read this submission and believe that I have an appropriate level of expertise to confirm that it is of an acceptable scientific standard.

# Response

**Published:** 2021-06-01

**Authors:** Mario Barbatti

**Affiliations:** 178807, France

We thank the reviewer for the report on our paper. Concerning the comment: “ *I encourage the authors to describe the further improvements they have made and, in case they have performed preliminary tests, perhaps they can describe their findings and, in this way, help other authors future attempts of implementations.*” At this moment, we still do not have any advances to report. Nevertheless, an advantage of publishing in a platform like Open Research Europe is that it is possible to update the paper to reflect new developments. We intend to report here any further methodological advances, as suggested by the reviewer.
